# Genomic and phenotypic characterization of two novel human-derived *Limosilactobacillus reuteri* strains with unique probiotic traits

**DOI:** 10.3389/fmicb.2025.1723084

**Published:** 2025-12-10

**Authors:** Lijuan Yang, Hongyu Zhan, Xiaoyu Wu, Aoqi Wang, Yi Zou, Jinjin Li, Yongjun Lu, Zhenhuang Ge

**Affiliations:** Runze Laboratory for Gastrointestinal Microbiome Study, School of Life Sciences, Sun Yat-sen University, Guangzhou, China

**Keywords:** comparative genomics, *Limosilactobacillus reuteri*, probiotics, strain isolation, strain-specificity

## Abstract

**Background:**

*Limosilactobacillus reuteri* is recognized for its diverse host-beneficial functions, though these are highly strain-specific. This study aimed to genomically and functionally characterize two novel *L. reuteri* isolates to assess their potential for specific health-associated applications.

**Methods:**

We isolated and genomically sequenced two novel *L. reuteri* strains, LLR2 and LLR3. Their functional potential was systematically evaluated through *in vitro* assays, including assessment of reuterin production, the ability to stimulate mucin production in co-culture with intestinal epithelial LS174T cells, and anti-inflammatory activity in a human brain microvascular endothelial cell (hCMEC/D3) model of LPS-induced inflammation.

**Results:**

Comparative genomic analysis revealed distinct genetic architectures between LLR2 and LLR3, reflecting divergent evolutionary trajectories. Functionally, pronounced strain-specific differences were observed: LLR3 produced the antimicrobial compound reuterin, while LLR2 did not. In co-culture with LS174T cells, both strains decrease the production of Mucin-2, a key component of the gut barrier, with LLR2 showing significantly greater efficacy. Conversely, in the hCMEC/D3 inflammation model, LLR3 demonstrated a significantly stronger anti-inflammatory effect, suppressing the LPS-induced expression of IL-6 and TNF-*α*.

**Conclusion:**

This study expands the genomic repository of *L. reuteri* and provides clear experimental evidence of strain-specific functional profiles. Our findings establish a foundation for the precision selection of *L. reuteri* strains such as LLR2 for gut barrier reinforcement and LLR3 for inflammation modulation in tailored applications for gut health and gut-brain axis research.

## Graphical abstract

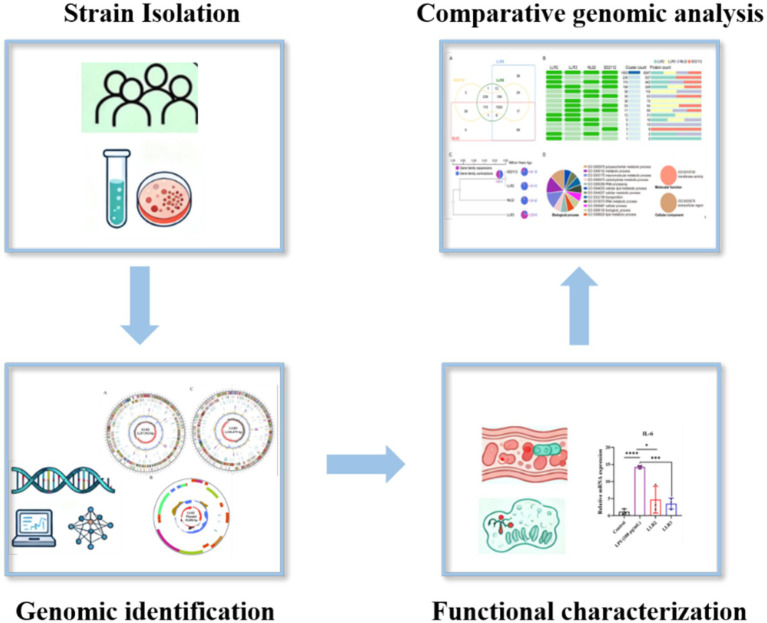

## 1 Introduction

Lactic acid bacteria are among the most widely used probiotics and are present in a variety of worldwide. As an important member of this group, different strains of *L. reuteri* exert probiotic functions through diverse mechanisms. For instance, JBD301 synthesizes short-chain fatty acids (SCFAs) to establish a chemical barrier (Chung et al., 2016). ATCC 55730 produces phenyllactic acid, exhibiting broad-spectrum antimicrobial activity against fungi and molds (Jørgensen et al., 2017). SBC5-3 generates signaling molecules such as histamine to modulate the host’s inflammatory response (Thomas et al., 2012). MJM60668 alleviates high-fat-diet-induced non-alcoholic fatty liver disease by inhibiting lipogenesis, promoting lipolysis, and synergizing with its anti-inflammatory activity to reduce hepatic lipid accumulation (Engevik et al., 2021). Pg4 expresses the *Clonostachys rosea* lactonohydrolase gene, enhancing its ability to remove zearalenone (Yang et al., 2017). Strain 12002 produces reuterin for meat decontamination and preservation (El-Ziney et al., 1999). SL001 improves growth performance, health-related parameters, intestinal morphology, and microbiota in broiler chickens (Chai et al., 2023). LR1 promotes growth, enhances feed utilization efficiency, prevents diarrhea, and regulates the immune system in pigs (Yi et al., 2018). Thus, these studies demonstrate that the functional properties of *L. reuteri* are highly strain-specific. Expanding the resource library of *L. reuteri* strains by isolating and identifying novel strains with enhanced or unique functional characteristics is essential for fully understanding and applying their probiotic potential. This will enable their utilization across various fields, including agriculture, animal husbandry, industrial fermentation, food processing, and pharmaceuticals.

Therefore, advancing *L. reuteri* applications necessitates a precision-based framework for selecting strains that combine robust antimicrobial activity with a well-defined probiotic profile. Genomics empowers the direct linkage of specific phenotypes to their genetic determinants, facilitating a critical transition from correlative observations to causal understanding (Huang et al., 2024). This paradigm shift allows us to decipher the diversity of probiotic functions from their genetic origins, thereby establishing an indispensable foundation for the ultimate goal of precision probiotic selection on the basis of genomic profiles.

The current repertoire of proprietary *L. reuteri* strains remains limited, highlighting the critical need for novel isolates with independent intellectual property. The establishment of a culturally independent strain collection through culturomics represents an essential foundation for advancing probiotic research and applications (Gangaiah et al., 2024; Li et al., 2025; Ong et al., 2019). This study contributes to this effort by isolating and characterizing two novel *L. reuteri* strains, thereby expanding the genetic resources available for subsequent investigations of strain-specific probiotic mechanisms and biotechnological development.

In this study, we systematically investigated the probiotic potential and antimicrobial properties of novel *L. reuteri* strains LLR2 and LLR3. Through comprehensive genome mining, we identified and annotated key genetic determinants involved in the biosynthesis of antimicrobial compounds, particularly those associated with reuterin production. The genomic insights were subsequently validated through experimental characterization of its antimicrobial activity and probiotic attributes. Furthermore, comparative genomic analyses against well-characterized *L. reuteri* strains revealed both conserved orthologous genes and strain specific genetic features. This integrated approach not only provides a foundation for the development of this strain as a novel probiotic agent but also provides valuable insights into the evolutionary adaptations of *L. reuteri* isolates.

## 2 Materials and methods

### 2.1 Strains, media, and culture conditions

*L. reuteri* strains LLR2 and LLR3 were isolated from the feces of a healthy adult. Species identification was performed through both morphological and molecular techniques. The strain was cultured in de Man, Rogosa and Sharpe (MRS) medium broth at 37 °C for 18 h under microaerophilic conditions. For long-term storage, the broth-cultured bacteria were mixed with 30% glycerol and preserved at −80 °C. This study was approved by the Internal Review Board and the Ethics Committee of the Sixth Affiliated Hospital of Sun Yat-sen University (2025ZSLYEC-393), and written informed consent was obtained from all volunteers.

### 2.2 Metabolite and enzyme activity analyses

*L. reuteri* strains LLR2 and LLR3 were cultured on solid media under anaerobic conditions for 16 h. Bacterial lawns were harvested and inoculated into MRS (Huankai Microbial, Cat. No. 027312) broth at an initial OD₆₀₀ of 0.05. For metabolite analysis, strains were cultured for 6 h to the mid-log phase and 0.4 OD units of bacterial cells were collected. For the culture experiments, strains were cultured for 16 h to stationary phase prior to harvesting.

#### 2.2.1 Metabolite analysis

Bacterial cultures were centrifuged at 10,000 × g for 10 min. For intracellular metabolites (GABA and GSH), cell pellets were resuspended in PBS and disrupted by ultrasonication. For extracellular metabolites (hyaluronic acid and *α*-glucosidase inhibition), supernatants were collected. Metabolite levels were quantified using commercial kits according to manufacturers’ protocols. *γ*-aminobutyric acid (GABA) concentrations were determined using a commercial ELISA kit (Cloud-Clone Corp., CEB022Ge) with PBS as blank control. Hyaluronic acid (HA) production was quantified via ELISA (Cloud-Clone Corp., CEA182Ge) using MRS broth as blank control. α-glucosidase inhibitory activity was measured with a specialized kit (Abnova, KA6219) against MRS broth blanks. Reduced glutathione (GSH) levels were assessed using a microassay kit (Nanjing Jiancheng, A006-2-1) with PBS blanks. Upon cultivation to stationary phase, cells were resuspended in anaerobic 250 mM glycerol containing 0.25% glucose and induce reuterin production at 37 °C lactose for 150 min. Reuterin production was determined colorimetrically. Briefly, a 10 mM L-tryptophan solution was prepared in 50 mM HCl, and a concentration gradient of 3-HPA from MCE (Cat. No. HY-N8461) standards was prepared in distilled water. Reaction mixtures containing 37.5 μL of tryptophan solution, 50 μL standards or test samples, and 150 μL concentrated HCl were incubated at 37 °C for 30 min. Absorbance was measured at 560 nm, and reuterin content was calculated against the standard curve after blank subtraction. The minimal inhibitory (MIC) concentrations of 3-HPA were determined using a microtiter assay based on the method described by Mota-Meira et al. (2005). We determined the MIC of purified reuterin against a panel of representative Gram-positive and Gram-negative bacteria. The tested strains included: Gram-negative: *Escherichia coli* and *Pseudomonas aeruginosa*. Gram-positive: *Staphylococcus aureus* and *Micrococcus luteus*.

#### 2.2.2 Enzyme activity assessment

Bacterial suspensions normalized to 10 OD₆₀₀ were concentrated (5,000 × g, 5 min) and resuspended in 200 μL MRS broth. Aliquots (3 μL) were spotted onto MP agar for protease activity and CMC agar for cellulase activity. Following 72 h anaerobic incubation at 37 °C, enzymatic activities were quantified by measuring clearance zones after appropriate staining.

#### 2.2.3 Co-culture systems with intestinal epithelial and brain microvascular endothelial cells

LLR2 and LLR3 strains were cultured under standardized conditions for co-culture experiments. For LS174T cell co-culture, strains were grown to mid-log phase (6 h cultivation) to ensure metabolic activity. For hCMEC/D3 cell co-culture, strains were cultured to stationary phase (16 h cultivation) to maximize metabolite production. Bacterial suspensions were normalized to 0.4 OD units prior to co-culture initiation. Both LS174T intestinal epithelial cells and hCMEC/D3 brain microvascular endothelial cells were maintained under optimal conditions. Cells were routinely subcultured using trypsin-based dissociation protocols with appropriate termination procedures. LS174T cells were cultured in EME medium supplemented with 10% FBS, while hCMEC/D3 cells were maintained in specialized endothelial cell medium. For experimental setups, cells from passage 2–3 were seeded into 12-well plates at 1 × 10^5^ cells/well and allowed to reach 75% confluence before co-culture initiation. Two distinct co-culture models were established via Transwell systems. For Mucin-2 studies with LS174T cells, bacterial suspensions (0.1 OD in 10% FBS-EME medium) were added to inserts in triplicate. For inflammation studies with hCMEC/D3 cells, cultures were pretreated with LPS (100 ng/mL) where indicated, followed by the addition of bacterial suspensions (0.1 OD in cell culture medium) to inserts. All co-cultures were maintained for 24 h under standard incubation conditions (37 °C, 5% CO₂). Following co-culture, supernatants and cells were collected for subsequent analysis. Mucin-2 secretion was quantified in LS174T co-culture supernatants using commercial ELISA kits. For hCMEC/D3 co-cultures, inflammatory responses were assessed through gene expression analysis of IL-6 and TNF-*α* via quantitative PCR following RNA extraction and reverse transcription.

### 2.3 Genome sequencing

The cryopreserved LLR2 and LLR3 strains stored at −80 °C were aseptically streaked onto MRS agar plates and subjected to anaerobic incubation overnight. Following the formation of isolated colonies, a single colony was inoculated into liquid MRS medium and cultured anaerobically overnight. The optical density at 600 nm (OD₆₀₀) of the bacterial suspension was measured, and subcultures were initiated at a starting OD₆₀₀ of 0.05 in fresh liquid medium. After 6 h of anaerobic cultivation, bacterial cells were harvested at the mid-logarithmic phase, corresponding to 10 OD units. The culture was centrifuged at 6,000 × g for 5 min, and the supernatant was discarded. The pellet was subsequently washed twice with phosphate-buffered saline (PBS), and genomic DNA from LLR2 and LLR3 strains was isolated employing a commercial DNA extraction kit (Omega Co.), following the manufacturer’s guidelines.

Samples were submitted to Beijing Biomarker Technologies Corporation for third-generation sequencing using Oxford Nanopore Technologies (ONT). All procedures were performed in strict accordance with the manufacturer’s standardized protocols, encompassing four main modules: sample quality control, library preparation, library quality assessment, and sequencing operation. The specific implementation steps were as follows:

Genomic DNA preparation and quality control: Nucleic acid purity was determined using a Nanodrop spectrophotometer, DNA concentration was quantified with a Qubit fluorometric system, and molecular integrity was evaluated by 0.35% agarose gel electrophoresis. Large-fragment DNA enrichment and purification: Nucleic acid fragments were selected and recovered using the BluePippin automated size selection system. Library preparation (SQK-LSK109 kit): DNA damage repair and end-modification were performed, followed by purification with magnetic beads. After adapter ligation, a second bead-based purification was conducted. Library quantification was carried out on the Qubit platform. Nanopore flow cell loading and sequencing run: Prepared libraries were loaded onto nanopore chips for sequencing.

### 2.4 Assembly and functional annotation

The complete genome sequence of *L. reuteri* was subjected to comprehensive bioinformatic analysis through an integrated workflow. Following initial quality control and *de novo* assembly with iterative error correction, structural annotation was performed using a suite of specialized tools: Prodigal for open reading frame prediction, tRNAscan-SE for tRNA identification, and Infernal v1.1 with the Rfam database for rRNA and non-coding RNA detection. Genomic features including CRISPR elements, prophages, and genomic islands were identified using CRISPR recognition tool, PhiSpy, and IslandPath-DIMOB, respectively. Functional annotation employed a multi-layered approach through homology searches against the NCBI Nr, SwissProt, TrEMBL, COG, and KEGG databases. Additional characterization included GO term assignment via Blast2GO, protein domain analysis with HMMER against Pfam, and specialized annotation using CAZyme, CARD, PHI, VFDB, and PathogenFinder databases. Secretory proteins were predicted through SignalP 6.0 analysis with transmembrane domain exclusion. Metabolic reconstruction was performed via RAST, while secondary metabolite gene clusters were identified using antiSMASH. Evolutionary relationships were elucidated through phylogenetic analysis in MEGA X and iTOL with complete *L. reuteri* genomes. All analyses were conducted using default parameters unless otherwise specified.

### 2.5 Comparative genomic analysis and evolutionary rate estimation

We performed multiple sequence alignment of *gyrB* sequences obtained from 21 complete *L. reuteri* genomes. Phylogenetic trees were constructed using the neighbor-joining method. To assess tree robustness, we conducted bootstrap analysis with 1,000 replicates. Based on phylogenetic relationships, we selected strains most closely related to LLR2 and LLR3 for further investigation. We retrieved general feature format files from the NCBI database. These files were used for pangenome analysis with a 95% identity threshold via megablast. Genomic synteny relationships were visualized using TBtools v1.09854 software with default settings. Orthologous gene clusters were identified using the OrthoVenn3 platform. The analysis parameters included an *E*-value cutoff of 1 × 10^−2^ and an inflation value of 1.5. This system employs graph-based clustering algorithms optimized for large-scale genomic datasets. For precise orthologous cluster definition, we conducted all-against-all protein sequence alignments using DIAMOND v0.9.24. The alignment threshold was set at BLASTX *E*-value <10^−3^. Gene Ontology annotation was performed through the AmiGO2 database. We calculated evolutionary rates by aligning orthologous genes between LLR2 and LLR3 strains. The computation included both nonsynonymous (Ka) and synonymous (Ks) substitution rates. Selective pressure was evaluated using the Ka/Ks ratio for each gene. Our classification criteria were as follows: Ka/Ks > 1 indicated strong positive selection, 0.5 < Ka/Ks ≤ 1 signified positive selection, 0.1 < Ka/Ks ≤ 0.5 represented weak positive selection, and Ka/Ks ≤ 0.1 corresponded to purifying selection. This analytical framework has proven effective for detecting positively selected genes in bacterial genomes (Ge et al., 2022).

## 3 Results and discussion

### 3.1 Comparative assessment of functional profiles in two *Limosilactobacillus reuteri* strains for targeted application

Given the well-documented strain-specific probiotic activities of *L. reuteri*, this study presents a systematic and comprehensive characterization of two distinct *L. reuteri* strains. Through in-depth profiling of their functional attributes, we aim to delineate their unique probiotic profiles and identify potential niche-specific applications.

#### 3.1.1 Microbial metabolites with probiotic effects

GABA, a nonproteinogenic amino acid widely distributed in microbes, plants and vertebrates, functions as a key inhibitory neurotransmitter in the mammalian central nervous system, where it regulates synaptic transmission, promotes neuronal differentiation and relaxation, and alleviates sleep and mood disorders (Tabouy et al., 2018). In addition to affecting the nervous system, GABA has diverse physiological activities in peripheral organs, including blood pressure regulation, glucose metabolism, tumor suppression, free radical scavenging, immune modulation, pathogen antagonism, allergy mitigation, and protection of the liver, kidney and intestine (Buffington et al., 2016; Connolly et al., 2016; Ngo and Vo, 2019). Using the ELISA detection kit, we found that the GABA concentrations in cell lysates of strains LLR2 and LLR3 were significantly higher than those of the control medium, reaching 35.04 pg/mL and 31.39 pg/mL, respectively, after 24 h of cultivation (Table 1). These results demonstrate that both strains possess the capacity to synthesize GABA. Hyaluronic acid (HA), which is valued for its viscoelasticity, lubricating function, and water-retention capacity, has broad applications in cosmetics, medicine, and biomedical engineering (Luis and Hansson, 2023). Using the CEA182Ge detection kit, we found that after 24 h of cultivation, the HA concentration in the fermentation supernatant of strain LLR3 (30.20 ng/mL) was nearly twice that of LLR2 (16.33 ng/mL), demonstrating the superior biosynthetic capacity of LLR3 (Table 1). Reduced glutathione (GSH), the most abundant endogenous thiol in mammalian systems, functions as a central nonenzymatic antioxidant through its free sulfhydryl group. As a *γ*-glutamyl-cysteinyl-glycine tripeptide, GSH eliminates reactive oxygen species, participates in detoxification and redox buffering, and supports diverse physiological processes including DNA synthesis, cell proliferation, immune regulation, and maintenance of redox homeostasis. Using biochemical assays, we found that both strains LLR2 and LLR3 produced comparable amounts of GSH, with concentrations of 91.09 μmol/L and 93.45 μmol/L, respectively, highlighting their potential contribution to antioxidant capacity (Table 1).

**Table 1:** Functional traits of *L. reuteri* LLR2 and LLR3 strains.

Function	LLR2	LLR3
GABA	35.04 pg/mL	31.39 pg/mL
Hyaluronic acid	16.33 ng/mL	30.20 ng/mL
Glutathione (GSH)	91.09 μmol/L	93.45 μmol/L
Diameter of proteolytic zone	2.44 ± 0.31 cm	1.95 ± 0.24 cm
Diameter of cellulolytic zone	0.774 ± 0.063 cm	—

**Figure 1:**
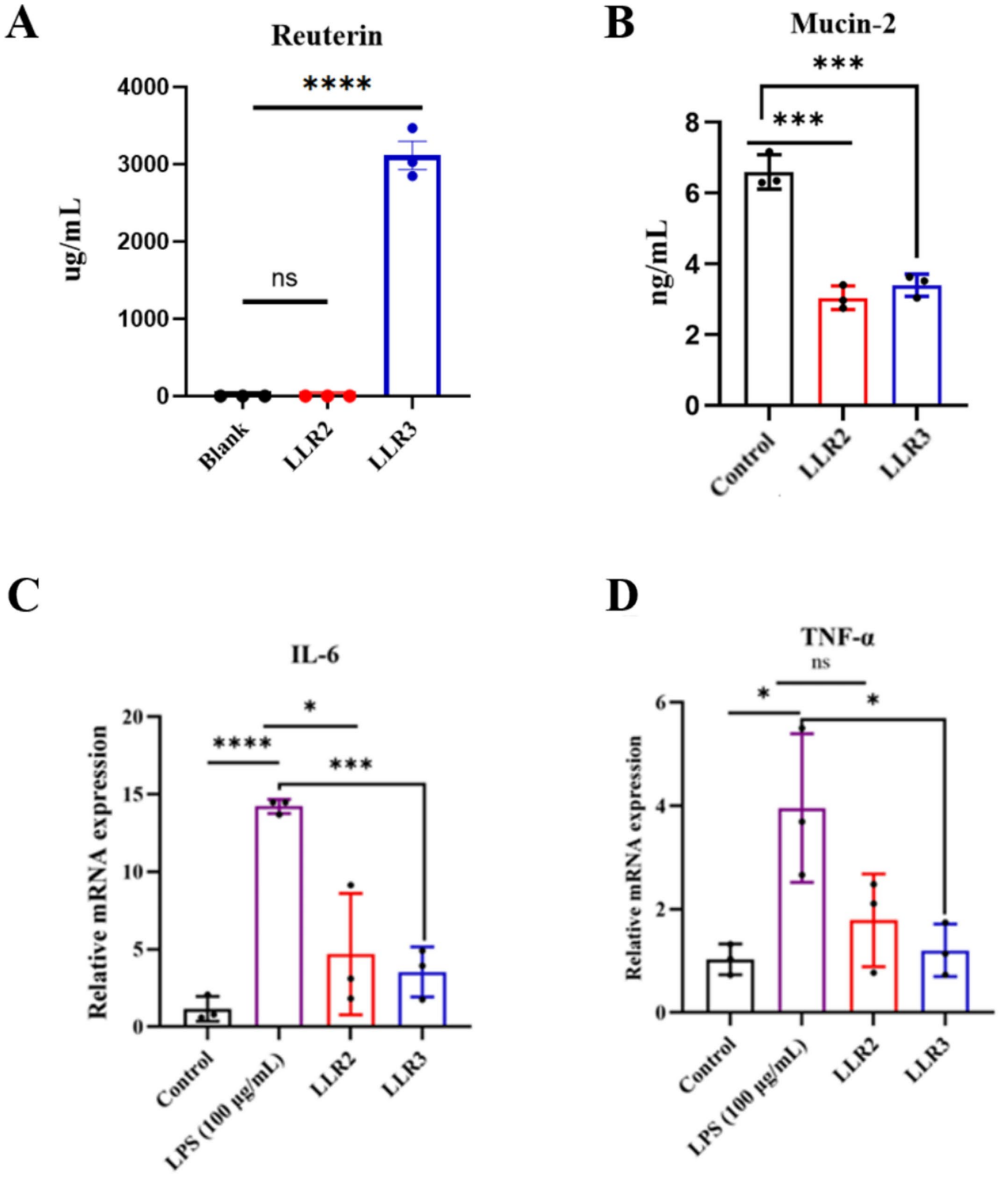
Functional traits of two *L. reuteri* strains. **(A)** Reuterin production by *L. reuteri* strains LL2 and LLR3. **(B)** Mucin-2 levels LS174T cells after co-culture with LLR2 and LLR3. **(C)** IL-6 expression levels in a human brain microvascular endothelial cell (HBMEC) after co-culture with LLR2 and LLR3. **(D)** TNF-*α* expression levels in HBMEC after co-culture with LLR2 and LLR3. ^ns^*p* > 0.05, ^*^p < 0.05, ^**^p < 0.01, ^***^p < 0.001, and ^****^p < 0.0001.

Reuterin, a metabolite produced by *L. reuteri* (Talarico et al., 1988), suggesting that it may act as a key effector molecule mediating the bacterium’s anticancer effects (Yenuganti et al., 2021). Notably, reuterin biosynthesis is strain-dependent and is limited to select human and avian-derived isolates. Reuterin comprises 3-hydroxypropionaldehyde (3-HPA) and its hydrated/ cyclized derivatives, with 3-HPA serving as a critical intermediate in glycerol conversion to 1,3-propanediol (Stevens et al., 2011). Its biosynthesis relies on glycerol dehydratase (GDH), a heterotrimeric complex of *α*, *β*, and *γ* subunits encoded by the co-localized pduCDE gene cluster (Cleusix et al., 2007; Greifová et al., 2017; Spinler et al., 2014). Whole-genome annotation revealed the presence of the pduCDE gene cluster in both LLR2 and LLR3, suggesting potential reuterin biosynthesis capability. Extracellular reuterin accumulation was quantified spectrophotometrically using a standard curve, with substrate solution serving as the baseline. As shown in Figure 1A, LLR3 produced substantial reuterin (3115.56 ± 477.1 μg/mL), whereas LLR2 exhibited no detectable production. Our functional assessment revealed that only LLR3, and not LLR2, demonstrated the capacity for reuterin production, confirming the functional specificity between these two strains. We determined the MIC of purified reuterin (produced and extracted from strain LLR3) against a panel of representative Gram-positive and Gram-negative bacteria. The tested strains included: Gram-negative: *Escherichia coli* (MIC, 7.26 ± 0.2 mM) and *Pseudomonas aeruginosa* (MIC, 0.42 ± 0.03 mM). Gram-positive: *Staphylococcus aureus* (MIC, 1.23 ± 0.14 mM) and *Micrococcus luteus* (MIC, 2.13 ± 0.56 mM). The results demonstrate that the reuterin produced by LLR3 exhibits potent, broad-spectrum antimicrobial activity.

#### 3.1.2 Enzymatic degradation by LLR2 and LLR3: utilization of proteins and cellulose

On milk protein agar (MP), both LLR2 and LLR3 produced diameters of Proteolytic zones, measuring 2.44 ± 0.31 cm and 1.95 ± 0.24 cm in diameter (Table 1), respectively, confirming their ability to secrete extracellular proteases. This proteolytic activity not only facilitates nutrient acquisition and ecological competitiveness but also may enhance host protein digestion and amino acid bioavailability, thereby contributing to probiotic functionality. Moreover, microbial proteases have potential applications in food processing and biotechnology, including the generation of bioactive peptides with health-promoting properties. Polysaccharide-degrading enzymes produced by gut commensals act in concert with host digestion to cleave plant-derived polysaccharides into soluble oligosaccharides, thereby enhancing nutrient bioavailability and modulating intestinal transit. Congo red staining revealed that LLR2, but not LLR3, formed a characteristic hydrolytic zone (0.774 ± 0.063 cm in diameter) on cellulose-containing plates, with a significantly stronger staining response than both the negative control and LLR3 (Table 1). These results demonstrate that LLR2 efficiently secretes extracellular cellulases and exhibits marked polysaccharide-degrading activity, a trait that may improve dietary fiber utilization and confer competitive advantages within the intestinal niche. Beyond ecological relevance, such enzymatic activity may also be harnessed in biotechnological applications, including the development of functional foods that target gut health.

#### 3.1.3 Intestinal barrier and inflammatory effects of *Limosilactobacillus reuteri* strains LLR2 and LLR3 in cellular models

The colonic mucus layer is structurally specialized, comprising an inner layer that is largely impenetrable to bacteria and an outer layer that expands to accommodate microbial colonization. Mucin-2, a heavily O-glycosylated glycoprotein secreted by goblet cells, is a key component of this mucus layer (Luis and Hansson, 2023). Certain members of the gut microbiota encode proteins capable of binding to and/or degrading mucins, and interactions between commensals and host mucins are critical for intestinal colonization. Upon treatment with LLR2 and LLR3, Mucin-2 levels in the cell supernatant were significantly reduced (Figure 1B), indicating that both strains can degrade Mucin-2. This mucin-degrading activity likely facilitates colonization within the mucus layer, enhancing microbial persistence and ecological fitness in the gut.

The gut-brain axis enables bidirectional communication between the central nervous system and the intestinal microbiota, with gut microbes acting as central modulators through metabolite-immune-neural pathways that influence nutrient absorption, energy homeostasis, neurotransmitter synthesis, and mood regulation (Aburto and Cryan, 2024; Luis and Hansson, 2023). Inflammation is a major driver of endothelial dysfunction, with IL-6 enhancing vascular permeability and TNF-*α* compromising the blood–brain barrier (Engevik et al., 2021). In a human brain microvascular endothelial cell (HBMEC) inflammation model induced by lipopolysaccharide (LPS), qPCR analysis showed that the expression of IL-6 was significantly elevated following LPS treatment, but co-culture with either LLR2 or LLR3 markedly reduced IL-6 levels (Figure 1C). Similarly, the expression of TNF-α was increased by LPS. While LLR2 had no significant effect, LLR3 co-culture significantly decreased TNF-α expression (Figure 1D). These results indicate that LLR3 can suppress both IL-6 and TNF-α, suggesting a potential role in restoring endothelial integrity and maintaining blood–brain barrier function under inflammatory conditions. The differential modulation of pro-inflammatory cytokines by LLR3 highlights its superior anti-inflammatory potential compared with LLR2. By attenuating key mediators of vascular permeability and blood–brain barrier disruption, LLR3 may contribute to neurovascular protection, positioning it as a promising candidate for microbiota-targeted interventions in neuroinflammatory or vascular disorders.

Additionally, certain strains of *L. reuteri* associated with human diseases have now entered the preclinical research and clinical validation stages, respectively (Supplementary Table S3). In preclinical research, strains such as CF48-3A, MM4-1A, and ATCC 6475 serve as powerful tools for mechanistic exploration in areas like oral pathogenic biofilms and neuroendocrine studies on the gut-brain axis. In contrast, clinically validated strains including DSM 17938 are now directly applied in practice to manage conditions such as infant colic. In this study, we report the isolation and initial characterization of the two novel *L. reuteri* strains. LLR3 demonstrated strong antibacterial activity, potentially mediated by bacteriocin production, along with marked anti-inflammatory properties. These attributes position LLR3 as a promising candidate for clinical prevention or adjunct therapy in inflammation-related conditions. In contrast, LLR2 exhibited distinctive capabilities in mucin degradation and extracellular enzyme activity. Given these metabolic traits, LLR2 appears better suited for food-based applications aimed at enhancing gut health and nutrient utilization.

Our findings illuminate a dual mechanism of action of LLR2 and LLR3, linking their local activity in the gut to systemic benefits for the brain. The observed mucin-utilization is not a pathobiont-like barrier breach but likely a symbiotic trigger for barrier reinforcement, a phenomenon where controlled breakdown stimulates robust regeneration. This enhanced gut barrier integrity may indirectly benefit brain health by reducing systemic inflammatory tone. More directly, the potent suppression of IL-6 and TNF-*α* in brain endothelial cells suggests a capacity of LLR3 to counteract central drivers of the dysfunction of Blood–Brain Barrier. The convergence of these pathways-fortifying the intestinal frontline while quelling cerebral inflammatory responses suggests that positions these strains, particularly LLR3, are compelling candidates for microbiota-based interventions. They exemplify the therapeutic concept of reinforcing the gut-brain axis at both ends, offering a promising strategy for managing conditions ranging from inflammatory bowel disorders to neuroinflammatory and vascular diseases.

### 3.2 Genome sequencing and assembly

Whole-genome sequencing revealed that LLR2 harbors a 2,217,922 bp circular chromosome and a 19,058 bp circular plasmid, whereas LLR3 consists of a single 2,181,079 bp circular chromosome (Table 2 and Supplementary Figure S1). The LLR2 chromosome has a GC content of 39.05% and encodes 2,143 genes, whereas its plasmid has a GC content of 36.86% and contains 20 genes. The LLR3 chromosome has a GC content of 38.84% and encodes 2,187 genes. Functional annotation further identified 69 tRNAs, 18 rRNAs, 2 CRISPR loci, 18 paralogous genes, 3 prophage regions, and 4 genomic islands in the LLR2 chromosome, compared with 70 tRNAs, 18 rRNAs, 4 CRISPR loci, 29 paralogous genes, 4 prophage regions, and 4 genomic islands in LLR3. These findings highlight both conserved and strain-specific genomic features between LLR2 and LLR3. The presence of a plasmid in LLR2 suggests potential for horizontal gene transfer and acquisition of accessory functions, whereas the higher number of CRISPR loci and prophage regions in LLR3 points to distinct evolutionary pressures and phage-host interactions. Together, these genomic differences may underlie functional divergence between the strains, shaping their ecological adaptation and probiotic potential (Table 2).

**Table 2:** General feature of LLR2 and LLR3 genomes.

Features	LLR2	pLLR2	LLR3
Type	Chromosome	Plasmid	Chromosome
Genome topology	Circular	Circular	Circular
Size (bp)	2,217,922	19,058	2,181,079
G + C content (%)	39.05	36.86	38.84
Number of genes	2,143	20	2,187
tRNA	69	0	70
rRNA	18	0	18
CRISPR number	2	0	4
Paralogous genes	18	0	29
Prophage	3	0	4
Genomic islands	15	0	14

### 3.3 Functional annotation

Functional annotation of the LLR2 and LLR3 genomic sequences was performed using multiple public databases, with results summarized in Supplementary Table S1. A total of 2,104 genes were functionally annotated, among which 988 genes had sequence lengths between 100–300 bp, and 865 genes exceeded 300 bp in length. In LLR2, a total of 2,158 coding genes were functionally annotated, among which 970 genes (44.9%) ranged between 100–300 bp in length, while 931 genes (43.1%) exceeded 300 bp. In LLR3, 2,164 coding genes were functionally annotated, with 1,027 genes (47.4%) falling within the 100–300 bp range and 844 genes (39.0%) longer than 300 bp.

**Figure 2:**
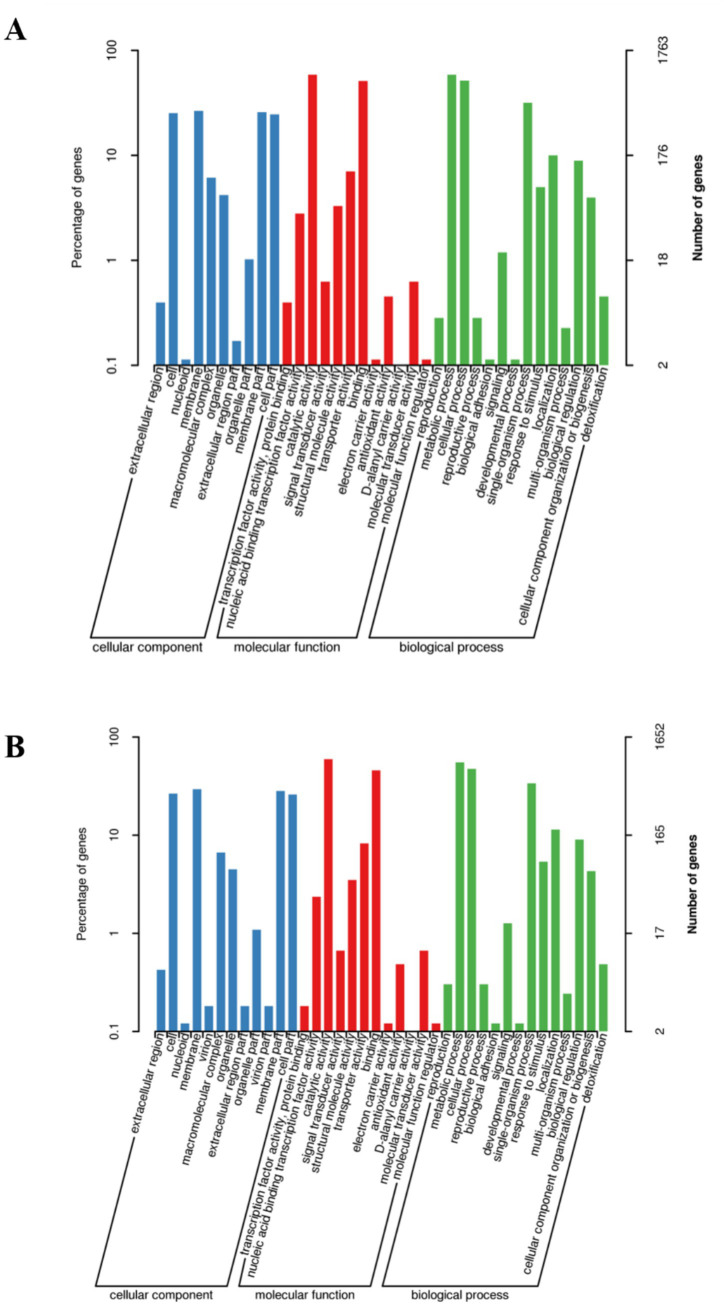
The result of classification statistics of GO pathway annotation of *L. reuteri*. **(A)** GO pathway annotation of *L. reuteri* LLR2. **(B)** GO pathway annotation of *L. reuteri* LLR3.

To functionally annotate the predicted genes, we conducted Gene Ontology (GO) and Kyoto Encyclopedia of Genes and Genomes (KEGG) enrichment analyses. GO consortium has established a standardized hierarchical vocabulary system for cross-species biomolecular functional characterization, with a continuously updated knowledge framework to accommodate scientific advances. Through the GO database, 2,162 genes in LLR2 and 2,186 genes in LLR3 were functionally annotated. As illustrated in Table 3 using genes encoding glycerol dehydratase as an example, LLR2 carries GE001602–GE001604 while LLR3 contains GE001861–GE001863, indicating that both strains possess glycerol dehydratase genes comprising three subunits. Based on the multi-level GO annotation system, genomic functional elements were systematically classified through hierarchical topology visualization, clearly demonstrating the distribution characteristics and regulatory network associations of different genetic units within specific functional subdomains. The annotated functions were categorized into three domains: Cellular Component, Molecular Function, and Biological Process. In LLR2, the predominant secondary classifications in Cellular Component included cell (444), membrane (468), membrane part (456), and cell part (434). For Molecular Function, catalytic activity (1,032) and binding (901) were most frequently annotated. Within Biological Process, metabolic process (1,037), cellular process (906), and single-organism process (561) represented the largest categories (Figure 2A). In LLR3, the major Cellular Component annotations were cell (439), membrane (485), membrane part (467), and cell part (429). For Molecular Function, catalytic activity (981) and binding (759) predominated. In Biological Process, the most abundant categories were metabolic process (911), cellular process (783), and single-organism process (559) (Figure 2B). KEGG pathway analysis revealed that the predicted genes of LLR2 were highly enriched in the pathways for purine metabolism and Biosynthesis of amino acids (Supplementary Figure S2).

**Table 3:** Part results of GO database annotation.

Strain	Gene_ID	GO_annotation
LLR2	GE001602	Cobalamin binding (GO:0031419); glycerol dehydratase activity (GO:0046405)
	GE001603	Propanediol dehydratase activity (GO:0050215)
	GE001604	Glycerol dehydratase activity (GO:0046405)
LLR3	GE001861	Glycerol dehydratase activity (GO:0046405)
	GE001862	Propanediol dehydratase activity (GO:0050215)
	GE001863	Cobalamin binding (GO:0031419); glycerol dehydratase activity (GO:0046405)

### 3.4 Antibiotic resistance

To evaluate the antibiotic resistance potential of LLR2 and LLR3, we performed genome-wide annotation using the Comprehensive Antibiotic Resistance Database (CARD), which integrates antimicrobial resistance ontology (ARO) with curated data on resistance determinants, molecular mechanisms, and drug-target interactions. Protein sequences were analyzed with the Resistance Gene Identifier (RGI) pipeline, enabling sensitive homology-based detection of conserved resistance motifs and inference of associated phenotypes and mechanisms. Notably, neither LLR2 nor LLR3 harbored identifiable resistance genes. The absence of detectable resistance determinants underscores the genomic safety of both isolates, strengthening their suitability for probiotic development and potential therapeutic application. Using PathogenFinder v1.1, the genomes of strains LLR2 and LLR3 were analyzed. No coding sequences belonging to pathogenic lineages were detected, indicating that LLR2 and LLR3 are not pathogenic to humans. These results also suggest that their ecological competitiveness and probiotic effects are unlikely to rely on antibiotic resistance mechanisms, further supporting their use in human health contexts.

### 3.5 Biosynthetic gene clusters of secondary metabolites

Microbial secondary metabolites represent crucial resources for developing antimicrobial agents and various bioactive compounds, encompassing clinically essential therapeutics such as conventional antibiotics, cholesterol-lowering agents, and anticancer drugs. To elucidate the biosynthetic mechanisms of these compounds, the research community widely employs antiSMASH v5.0.0 as a core analytical tool. This platform is specifically designed to identify functional units encoding bioactive molecule synthesis in bacterial and fungal genomes. The algorithm integrates characteristic sequence databases of genomic modules associated with different classes of metabolite synthesis, combined with hidden Markov model (HMM)-based intelligent recognition technology, to precisely locate genetic coding regions involved in the production of diverse chemical structures of secondary metabolites. Beyond basic localization functionality, the tool also performs in-depth sequence feature analysis, including domain composition and conserved motif identification. Both LLR2 and LLR3 contain a large gene cluster spanning 41 kb, though LLR2’s cluster comprises 43 genes while LLR3’s contains 38 genes (Supplementary Table S2).

### 3.6 Subcellular localization analysis of proteins

#### 3.6.1 Signal peptides

Signal peptides are short N-terminal sequences synthesized during translation that act as molecular addresses, directing nascent polypeptides to the appropriate membrane system. Following recognition by the signal recognition particle (SRP) complex and translocation via membrane transport machinery, the leader peptide is cleaved by signal peptidase, enabling maturation of the functional protein. In this study, secretory signal sequences were predicted using SignalP v6.0 (Petersen et al., 2011), which employs machine learning-based models to evaluate the first 70 amino acids of each protein for compositional patterns, hydrophobicity, and conserved cleavage motifs. The analysis identified 67 signal peptides in LLR2 and 82 in LLR3 (Table 4). The larger repertoire of signal peptides in LLR3 suggests an expanded capacity for protein secretion compared with LLR2, consistent with its predicted higher number of extracellular proteins. These findings indicate that strain-specific differences in secretory machinery may underpin variations in ecological fitness, host interactions, and probiotic functionality.

**Table 4:** Results of protein subcellular localization analysis.

Strain	Protein type	Number
LLR2	Signal peptide	67
	Transmembrane protein	478
	Secreted protein	33
LLR3	Signal peptide	82
	Transmembrane protein	524
	Secreted protein	40

#### 3.6.2 Transmembrane helices

Integral membrane proteins are essential components of biological membranes, and are typically characterized by one or more *α*-helical transmembrane domains spanning the phospholipid bilayer, coupled with cytoplasmic or extracellular domains that mediate functional interactions. Topological analysis of such proteins was performed using TMHMM v2.0, which applies a hidden Markov model to accurately identify transmembrane helices and delineate soluble-membrane boundary coordinates. This analysis predicted 478 transmembrane proteins in LLR2 and 524 in LLR3 (Table 4). The substantial number of transmembrane proteins in both strains underscores their complex membrane-associated functional repertoire, including roles in nutrient uptake, signal transduction, stress adaptation, and host–microbe interactions. The slightly larger set identified in LLR3 may reflect enhanced metabolic versatility and environmental adaptability, potentially contributing to its distinct probiotic traits compared with LLR2.

#### 3.6.3 Secreted proteins

Extracellular secreted proteins, which are released via dedicated transport pathways, represent key microbial effectors. They include hydrolytic enzymes that depolymerize complex biomolecules such as proteins and polysaccharides into absorbable oligomers, thereby enhancing ecological adaptability, as well as antimicrobial peptides (e.g., bacteriocins) that mediate competitive interactions within microbial communities. At the mechanistic level, polypeptides carrying N-terminal signal peptides are typically directed to the membrane or secretory system via the signal recognition particle (SRP)-dependent pathway. To identify putative secreted proteins, we combined SignalP-based signal peptide predictions with TMHMM-based transmembrane topology analyses, excluding membrane-anchored peptides through differential filtering. This analysis identified 33 secreted proteins in LLR2 and 40 in LLR3 (Table 4). The predicted secretomes highlight both shared and strain-specific extracellular functional capacities. The slightly larger repertoire in LLR3 may indicate broader metabolic versatility and competitive potential, consistent with its observed functional traits. These proteins likely contribute to nutrient acquisition, ecological adaptation, and inter-microbial interactions, and may represent important determinants of probiotic efficacy.

### 3.7 Comparative genomic analysis

**Figure 3:**
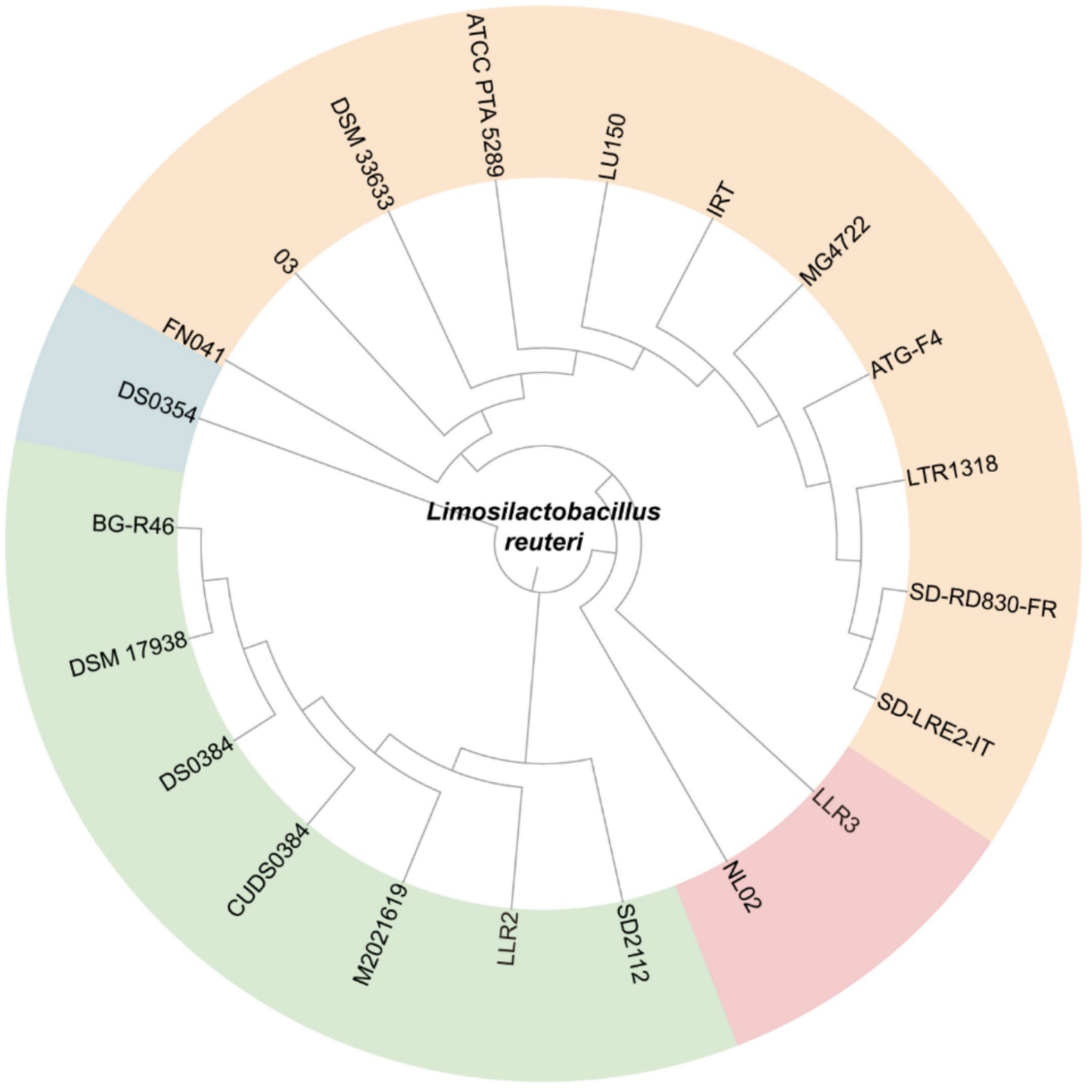
Neighbor-joining method phylogenetic tree of *L. reuteri* LLR2 and LLR3 based on *gyrB* gene sequences. This analysis involved 21 nucleotide sequences from all complete genome sequenced *L. reuteri* strains using the neighbor-joining method. Green branches represent strains most closely related to LLR2 and LLR3.

To evaluate the phylogenetic relationships and genetic diversity of LLR2 and LLR3 among related strains, a maximum-likelihood phylogenetic tree was constructed based on 16S rRNA sequences using MEGA X (Han et al., 2024). The analysis included a comparison of 23 complete genomes of human-derived *L. reuteri* retrieved from the GenBank database, positioning LLR2 and LLR3 within the full phylogenetic context of the species. The branches of *L. reuteri* LLR3, LLR2, SD2112, and NL02 formed a subclassification, with LLR2 being the closest to LLR3 (Figure 3). Subsequently, a whole-genome comparative genomic analysis was conducted among these four strains using TBtools software (Chen et al., 2020), with LLR3 as the reference genome. Synteny blocks among the four *L. reuteri* strains LLR2, LLR3, SD2112, and NL02 were identified. The results showed that there were 2,654, 2,757, and 2,687 collinear blocks between the pairwise comparisons of LLR3 vs. LLR2, LLR3 vs. NL02, and LLR3 vs. SD2112, respectively. These results indicate that the LLR2 genome exhibits a higher degree of syntenic conservation with LLR3 than with SD2112, and NL02 (Supplementary Figure S3).

**Figure 4:**
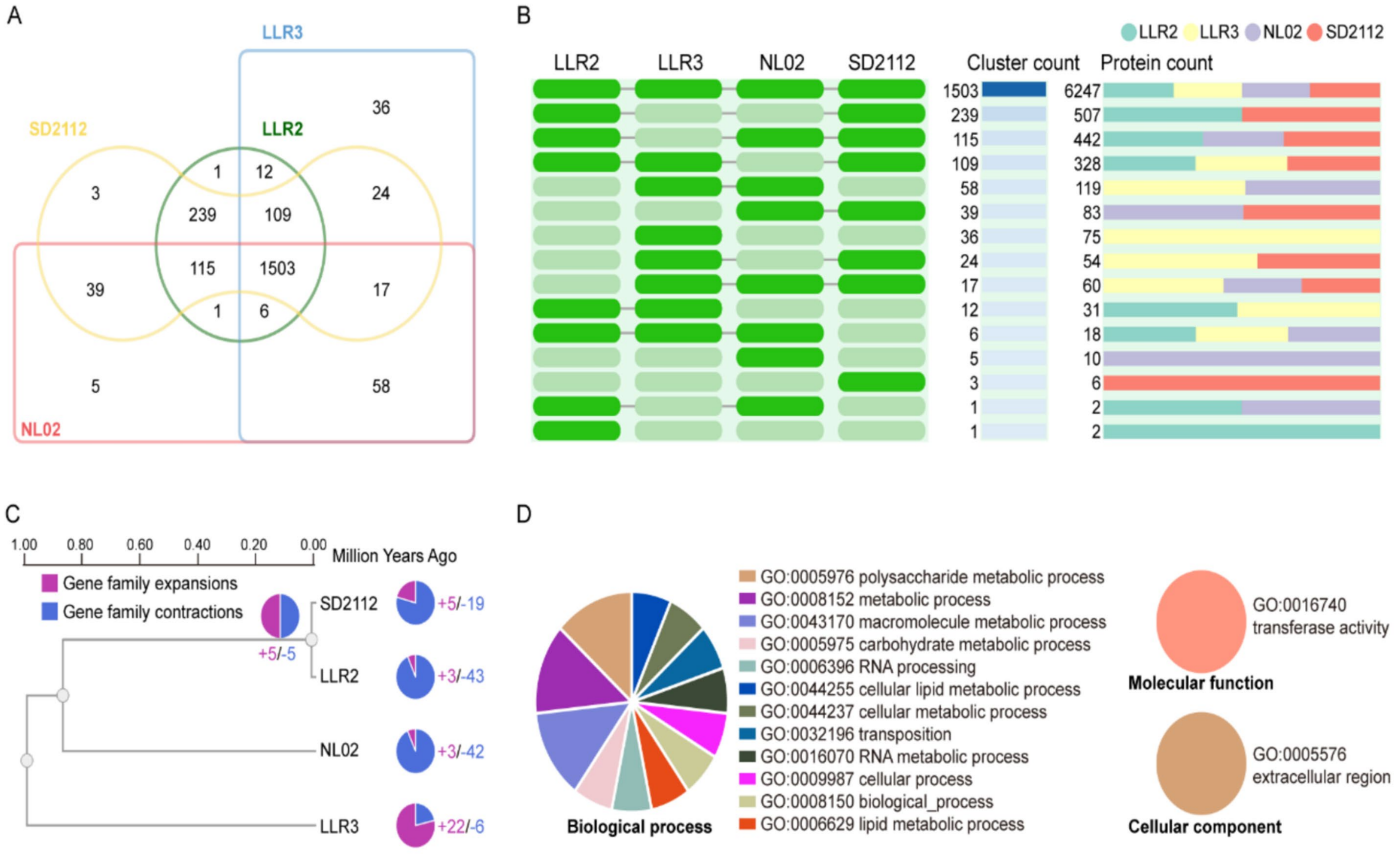
Comparison and annotation of orthologous gene clusters among *L. reuteri* LLR3, LLR2, SD2112, and NL02. **(A,B)** Venn diagram showing the shared/unique orthologous gene cluster numbers of strain *L. reuteri* LLR3, LLR2, SD2112, and NL02. A green cell represents the presence of a cluster group in the corresponding species, and a gray cell signifies its absence. **(C)** Dynamics of gene family evolution. **(D)** The biological processes of LLR3 specific gene clusters, molecular function, and cellular component of LLR3 specific gene clusters.

To elucidate gene and genome evolution patterns, we performed comparative analysis of whole-genome orthologous clusters among *L. reuteri* strains LLR2, LLR3 and reference strains. The analysis identified 1,509 core genome orthologs (Figures 4A,B) identified in LLR3, among which 1,618 orthologous gene clusters corresponding to the similarities between protein sequences from the LLR2 vs. NL02, and 1,584 orthologous gene clusters corresponding to the similarities between protein sequences from LLR3 and NL02. In microbial evolution, the core drivers of such lineage diversification are often linked to niche adaptation and the differential acquisition of horizontal gene transfer. LLR3 exhibit significant gene family expansion, which typically originates from the acquisition of specific genomic islands or events associated with plasmids and bacteriophages, leading to a gene dosage effect. This expansion may directly enhance their adaptive advantage in specific environments, for instance, by amplifying virulence factors, secondary metabolite gene clusters, or stress resistance genes to achieve functional radiation. Conversely, lineages that undergo massive gene family contraction LLR2 and NL02, typically follow a path of reductive evolution, genomes undergo significant streamlining and optimization by shedding non-essential metabolic pathways and regulatory genes in stable, nutrient-rich environments, thereby increasing their replication efficiency (Figure 4C). This evolutionary pattern is a classic manifestation of adaptive gene loss. Therefore, the pronounced disparities in gene family dynamics among different microbial lineages profoundly reflect their distinct life-history strategies and niche specificity. Expansion serves as an innovative engine for microbes to exploit new ecological spaces, while contraction represents a path to refinement and high specialization in stable environments.

To functionally contextualize these strain-specific genetic features, we conducted Gene Ontology (GO) (Ashburner et al., 2000) enrichment analysis. GO enrichment analysis further showed these strain-specific genes are significantly enriched in biological processes including polysaccharide metabolic process (GO:0005976), metabolic process (GO:0008152), macromolecule metabolic process (GO:0043170), carbohydrate metabolic process (GO:0005975), RNA processing (GO:0006396), transposition (GO:0032196), and various cellular processes (GO:0044255/GO:0044237/GO:0009987). Molecular function analysis revealed significant enrichment in transferase activity (GO:0016740) and Cellular component analysis revealed significant enrichment in extracellular region (GO:0005576) (Figure 4D).

**Figure 5:**
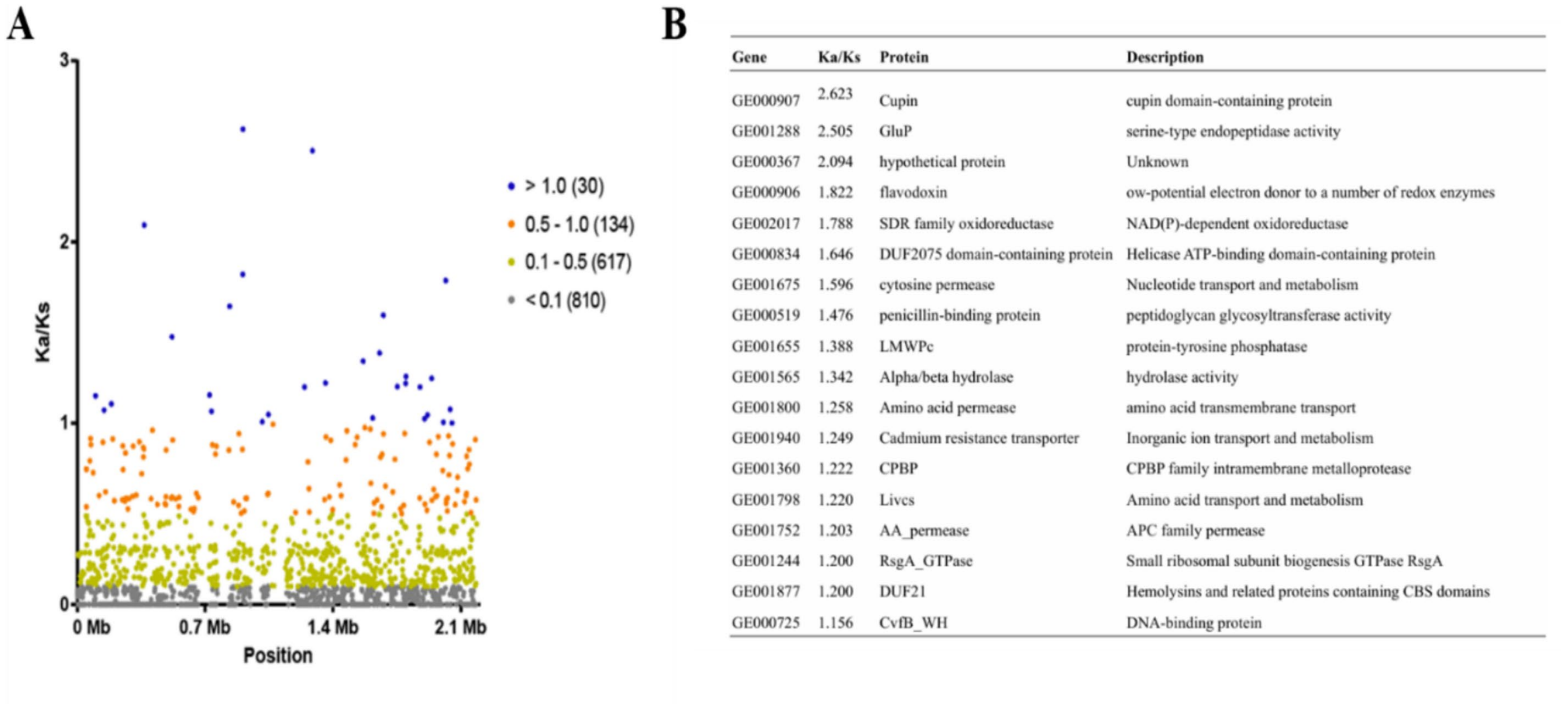
Substitution rate of orthologous genes in LLR3 compared with LLR2. **(A)** The substitution rate (Ka/Ks) of one-to-one orthologous genes in LLR3 compared with in LLR2. **(B)** The strong positively selected genes between LLR2 and LLR3.

The ratio of nonsynonymous to synonymous substitutions (Ka/Ks) in single-copy orthologous genes serves as an established metric for evaluating selective pressure during evolution (Hurst, 2002). To assess evolutionary dynamics in *L. reuteri*, we computed Ka/Ks ratios for orthologous genes across selected strains using the TBtools Ka/Ks calculator. Between LLR2 and strain LLR3, analysis of 1,591 orthologous gene pairs identified 30 under strong positive selection (Ka/Ks > 1.0), 134 under positive selection (0.5 < Ka/Ks ≤ 1.0), 617 under weak positive selection (0.1 < Ka/Ks ≤ 0.5), and 810 under purifying selection (Ka/Ks ≤ 0.1) (Figure 5A). Genes in LLR3 under strong positive selection predominantly encode proteins including Cupin domain-containing protein, Rhomboid family intramembrane serine protease, flavodoxin, SDR family oxidoreductase, DUF2075 domain-containing protein, cytosine permease, penicillin-binding protein, LMWPc, alpha/beta hydrolase, amino acid permease, cadmium resistance transporter, CPBP, branched-chain amino acid transport system II carrier protein, AA_permease and hypothetical protein (Figure 5B). The identification of genes under strong positive selection in LLR3 provides valuable insights into its adaptive evolution and potential probiotic mechanisms. The marked enrichment of transport-associated genes, such as cytosine permease, cadmium resistance transporter, branched-chain amino acid carrier, and multiple amino acid permeases points to significant evolutionary optimization for efficient nutrient acquisition and detoxification capacity. Such a specialized transport system is likely instrumental in enhancing its ecological fitness and competitiveness within the complex intestinal niche. In addition to nutrient utilization, the detection of rhomboid family intramembrane serine protease and alpha/beta hydrolase under strong selection suggests refined regulatory capacity in protein processing and signal modulation. These enzymes may facilitate host-microbe crosstalk through the remodeling of surface-associated proteins or the generation of bioactive molecules. The simultaneous selection of these functionally diverse genes reflects LLR3’s multi-dimensional adaptation strategy, balancing nutrient acquisition, stress resistance, and host interaction attributes that collectively increase its potential as a probiotic candidate for gut health applications.

## 4 Conclusion

This study provides a comprehensive genomic and functional characterization of two novel *L. reuteri* strains, LLR2 and LLR3, significantly expanding the genomic resources for this important probiotic species. Our analyses revealed remarkable strain-specific differences in genomic architecture and functional capabilities: LLR3 demonstrated robust antibacterial and anti-inflammatory activities, likely mediated by reuterin and bacteriocin production, whereas LLR2 exhibited unique advantages in mucin utilization and extracellular enzyme activity. These traits, encoded by specialized gene clusters related to bacteriocin synthesis, transmembrane transport, and stress adaptation, underscore the functional diversification within the species. Importantly, we demonstrated that even strains isolated from the same geographic environment by the same investigator can diverge at both evolutionary and functional levels. This offers a critical insight for microbiota research, seemingly similar microbial communities across individuals may exert distinct effects due to strain-level variation. Consequently, a strain-oriented approach, guided by detailed genetic information, is essential to advance our functional understanding and enable tailored microbial interventions. The genomic and functional framework established here will support the rational selection and optimization of *L. reuteri* for targeted applications in food and healthcare, guiding future mechanistic and translational studies.

## Data Availability

The complete genome sequences of Limosilactobacillus reuteri strains LLR2 and LLR3 have been deposited in the NCBI database with the following accession numbers: BioProject PRJNA1371660 and BioSample SAMN53506833 for LLR2; BioProject PRJNA1371670 and BioSample SAMN53509514 for LLR3.
