# Supplementary material for: Genomic and phenotypic characterization of two novel human-derived Limosilactobacillus reuteri strains with unique probiotic traits
Source: Front Microbiol. 2025 Dec 10;16:1723084. doi: 10.3389/fmicb.2025.1723084 (PMC12728014; doi:10.3389/fmicb.2025.1723084)
Supplement: Supplementary file 1 [file Table_1.DOCX]

**Supplementary information**


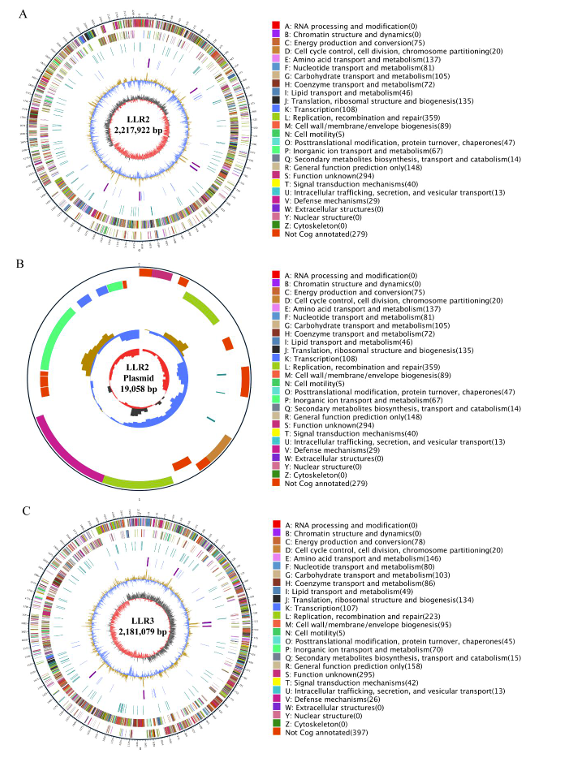


**Figure S1** Whole genome of *Limosilactobacillus reuteri* LLR2 and LLR3. The genome is depicted as six concentric circles, denoting (from outer to inner): the genomic size (5 kb per interval), CDSs on the forward strand, CDSs on the reverse strand, tRNA (blue) and rRNA (purple) genes, GC content, and GC skew.

**
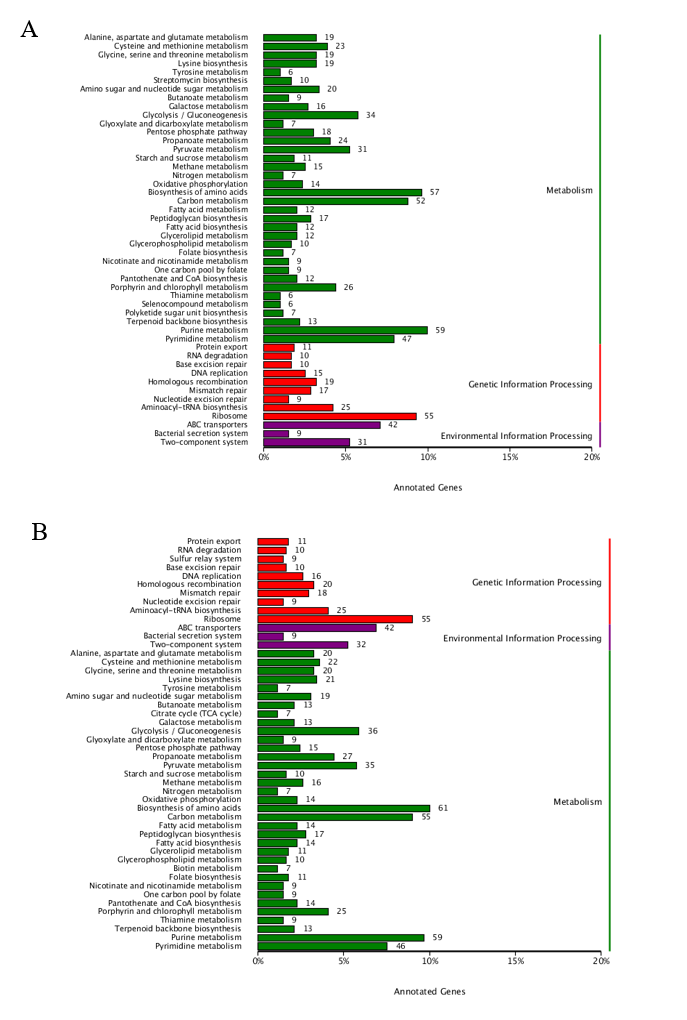
**

**Figure S2** The result of classification statistics of KEGG pathway annotation of *Limosilactobacillus reuteri* LLR2 (A) and LLR3 (B).


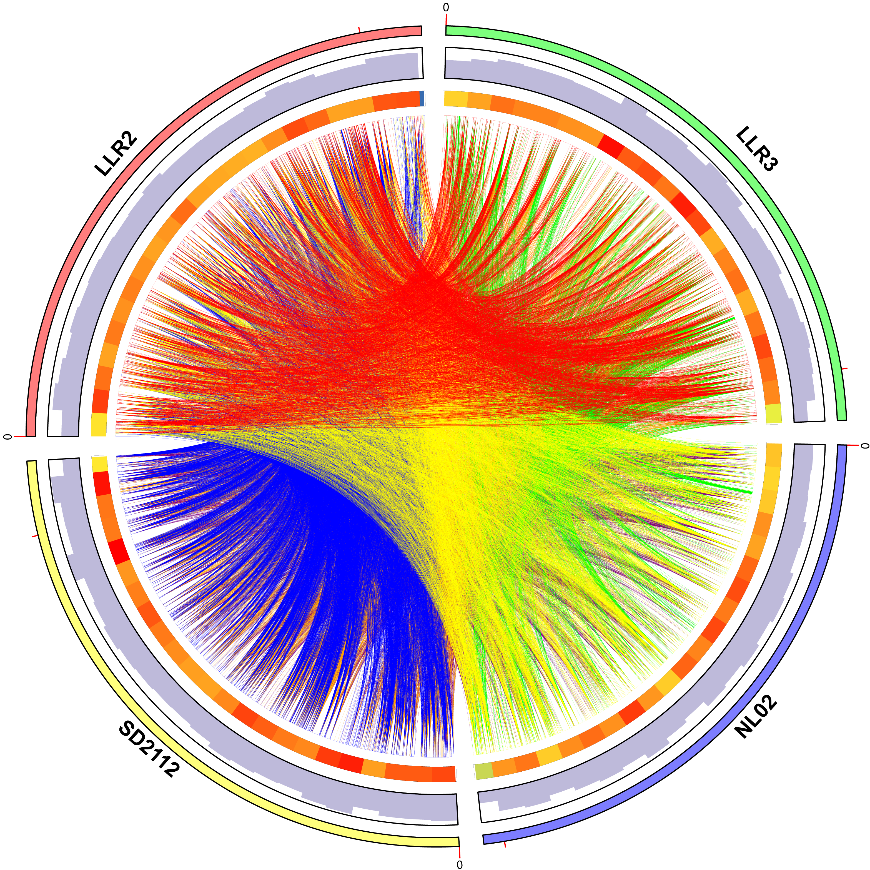


**Fig. S3 Synteny map of *L. reuteri* strains LLR2, LLR3, SD2112 and NL02 genomes.** Synteny map of *Lactobacillus reuteri* LLR2, LLR3, SD2112 and NL02 genomes. Genomic comparison of *L. reuteri* LLR3 strain against closely related representative *L. reuteri* LLR2, SD2112 and NL02. From outer to inner: genome labels, gene frequencies, and intragenomic collinearity blocks (colored lines).

**Table S1**

**General Database Statistics** **of *Limosilactobacillus reuteri* LLR2 and LLR3.**

| **ID** | **LLR2** | | | **LLR3** | | |
| --- | --- | --- | --- | --- | --- | --- |
| Database | Number | 100<Length<300 | Length>=300 | Number | 100<Length<300 | Length>=300 |
| eggNOG_Annotation | 1884 | 846 | 906 | 1790 | 854 | 810 |
| GO_Annotation | 1763 | 809 | 812 | 1652 | 784 | 723 |
| KEGG_Annotation | 1087 | 476 | 550 | 1104 | 490 | 546 |
| Nr_Annotation | 2157 | 970 | 931 | 2159 | 1026 | 844 |
| Pfam_Annotation | 1848 | 822 | 899 | 1776 | 847 | 805 |
| Swissport_Annotation | 1323 | 514 | 746 | 1209 | 502 | 644 |
| TrEMBL_Annotation | 2156 | 970 | 931 | 2152 | 1022 | 844 |
| All_Annotation | 2158 | 970 | 931 | 2164 | 1027 | 844 |

**Table S2**

**Prediction of gene clusters of *Limosilactobacillus reuteri* LLR2 and LLR3.**

| ID | Gene_cluster | Start | End | Length(bp) | Gene number |
| --- | --- | --- | --- | --- | --- |
| LLR2 | r1c1 | 610.595 | 651,752 | 41,158 | 43 |
| LLR3 | r1c1 | 615,241 | 656,398 | 41,158 | 38 |

**Table S3 Comparative overview of *L. reuteri* strains**

| Study | Strain | Source / Origin | Chromosome (bp) | Reuterin | Key Functions | Mechanism |
| --- | --- | --- | --- | --- | --- | --- |
| Clinical study | DSM 17938 | Human breast milk | 2,223,332 | Yes | Gastrointestinal health of infants and young children | Infant colic, general gut health |
|  | ATCC PTA 6475 | Human | 2,067,914 | Yes | Skin health and metabolism | Immune regulation and neuroendocrine regulation (oxytocin) |
|  | ATCC PTA 5289 | Human | 2,039,512 | Yse | Oral health | Produce reuterin and competitive exclusion |
| Preclinical study | CF48-3A | Gastrointestinal_tract | 2,107,903 | Yes | Efficiently inhibit oral pathogens | It effectively produces reuterin and regulates biofilms |
|  | MM4-1A | Gastrointestinal_tract | 2,067,914 | Yes | Improve oral health | Produce reuterin and enhance the barrier |
|  | ATCC PTA 4659 | Breast milk | 2,096,828 | Yes | Inhibit Helicobacter pylori | Induce oxytocin and regulate the HPA axis |
| This study | LLR2 | Human | 2,217,922 | No | Intestinal barrier | Mucin utilizes mucin and extracellular enzyme activity |
|  | LLR3 | Human | 2,181,079 | Yes | Anti-inflammatory and antimicrobial roles | Produces reuterin and anti-inflammatory effects |
